# Prolonged Postoperative Paralysis Following Elective Tonsillectomy: A Case of Pseudocholinesterase Deficiency

**DOI:** 10.7759/cureus.98727

**Published:** 2025-12-08

**Authors:** Kade Walsh, Shane Smith, Gabriella Manilla, Jatin Ahluwalia, Robert Stachler

**Affiliations:** 1 Otolaryngology - Head and Neck Surgery, Rocky Vista University College of Osteopathic Medicine, Parker, USA; 2 Anesthesiology, Rocky Vista University College of Osteopathic Medicine, Parker, USA; 3 Otolaryngology - Head and Neck Surgery, Ascension Providence Hospital, Southfield, USA; 4 Otolaryngology, Beaumont Hospital, Royal Oak, USA

**Keywords:** anesthesia complications, delayed emergence from anesthesia, pseudocholinesterase deficiency, succinylcholine, tonsillectomy

## Abstract

Pseudocholinesterase deficiency is a rare disorder of the enzyme butyrylcholinesterase that can lead to delayed emergence following exposure to depolarizing neuromuscular blocking agents. We report the case of a 19-year-old male who underwent elective tonsillectomy and experienced prolonged postoperative paralysis, suspected to be due to pseudocholinesterase deficiency. The patient had no significant past medical history or family history of the condition. This report highlights the clinical implications of pseudocholinesterase deficiency and discusses strategies for perioperative management.

## Introduction

Pseudocholinesterase deficiency is a disorder that can lead to unexpected perioperative complications and has profound patient safety implications. Complications can be characterized by apnea and prolonged paralysis following administration of depolarizing neuromuscular blocking agents [1]. The disorder can be acquired or genetic, with the genetic form differentiated into either homozygous or heterozygous forms [1]. The inheritance pattern is autosomal recessive, and is caused by mutations in the *butyrylcholinesterase* (*BCHE*) gene on chromosome 3q26.1-q26.2 [1]. Approximately 1 in 3,200 individuals to 1 in 5,000 individuals are estimated to have the disorder in the general population [2]. The acquired form may be attributed to medication use, pregnancy, liver disease, burns, or malnutrition [2,3]. Interestingly, recent literature suggests that Iranian Jews may express the atypical allele at a higher frequency (12.1% heterozygous, 0.9% homozygous) relative to the general population [4]. Although genetic forms are present at birth, the average age of diagnosis varies significantly, depending on when the individual is exposed to an inciting muscle relaxant [1]. Therefore, most cases of pseudocholinesterase deficiency arise perioperatively when a patient presents with unexpectedly prolonged paralysis and apnea, as well as delayed overall recovery from anesthesia [1]. Because most patients are asymptomatic until exposed to an offending agent, anesthesiologists and surgeons alike must maintain awareness of this condition to anticipate and manage unexpected delayed emergence.

The most common inciting agents include ester-based neuromuscular blocking agents such as succinylcholine and mivacurium [5]. Ester-based local anesthetics such as chloroprocaine and procaine have also been noted to have prolonged effects [6]. These drugs are all metabolized via plasma cholinesterases, primarily butyrylcholinesterase in humans [5]. Butyrylcholinesterase is also referred to as pseudocholinesterase or plasma cholinesterase. The enzyme itself is produced in the liver and released into circulation, where it exerts its effects [7].

Under normal conditions, there is a sufficient supply of plasma butyrylcholinesterase to rapidly hydrolyze these agents, resulting in safe, predictable anesthetic dosages. However, in individuals with pseudocholinesterase deficiency, hydrolysis occurs at a far slower rate, resulting in prolonged skeletal muscle paralysis [7]. It is important to note that acetylcholinesterase is not responsible for the metabolism of these drugs in the plasma and is restricted to the neuromuscular junction [8].

## Case presentation

A 19-year-old caucasian male with a history of recurrent tonsillitis presented to our hospital for elective tonsillectomy. He had no previous surgeries or chronic medical conditions. Additionally, he had no documented medical or seasonal allergies. He denied the use of alcohol, tobacco, or other illicit drugs. He also denied any known family history of anesthetic-related complications, including malignant hyperthermia and pseudocholinesterase deficiency.

On the day of the procedure, the patient was seen and examined in the preoperative area. The patient weighed 79.4 kg, had a body mass index of 26 kg/m², and an American Society of Anesthesiologists status of 2. His blood pressure preoperatively was 133/72 mmHg, and his respiratory rate was within normal limits at 16 breaths/minute. Physical examination was significant for bilateral tonsillar hypertrophy (graded as 3+/4 bilaterally) with the presence of tonsillar crypts bilaterally. He did not have evidence of tonsilliths, exudates, uvular deviation, or trismus on the day of surgery. Informed verbal consent to proceed with the procedure was obtained.

The patient was brought to the operating room and received standard anesthetic induction for the facility. The patient was induced with fentanyl (100 µg), propofol (180 mg), and succinylcholine (100 mg). He was intubated using direct laryngoscopy, and an oral Ring-Adair-Elwyn tube was placed without complication. This is the standard induction medication and endotracheal tube used at this facility for patients undergoing tonsillectomy. Throughout the entirety of the case, anesthesia was maintained with 100 µg of fentanyl, 400 mg of propofol, and sevoflurane inhaled anesthetic. Succinylcholine was not administered following induction. All vital signs remained within normal limits. The surgery was uncomplicated, and upon completion, care was resumed by the anesthesia team for extubation. Approximately 30 minutes after all anesthetic agents were discontinued, the patient remained apneic and could not be extubated. At this time, the anesthesia team proceeded to use acceleromyography to assess neuromuscular blockade, and continued paralysis was confirmed. He was observed for an additional 10 minutes. Upon discussion with multiple anesthesia team members, it was deemed most likely that the patient had a pseudocholinesterase deficiency as he remained paralyzed for nearly 60 minutes after receiving a one-time dose of succinylcholine. A comprehensive metabolic panel was also drawn to rule out hyperkalemia. All laboratory values were within normal limits. At this time, it was decided to admit the patient to the intensive care unit for continued observation, as the patient would need to remain intubated. He was sent to the intensive care unit in stable condition and maintained on total intravenous anesthesia. In the intensive care unit, the patient remained stable. The patient was progressively weaned off sedation. He was noted to have a return of spontaneous breathing approximately five hours after administration of succinylcholine, and he was extubated to room air without complication in the intensive care unit. The patient was discharged home in stable condition. Upon further discussion with the patient’s family, it was confirmed from the patient’s parents that there was no known family history of pseudocholinesterase deficiency. Both his mother and father had previous surgeries requiring general anesthesia, and they reported no complications with anesthesia. Following discharge, the patient was seen at his postoperative visit approximately one week after surgery. At that time, the bilateral tonsillar fossae were noted to be healing appropriately. The patient denied any long-lasting symptoms related to receiving general anesthesia, including nausea, headache, muscle aches, or excessive fatigue. At this one-month postoperative visit, the patient’s oropharynx appeared to be well healed, and he reported adequate patient satisfaction following the procedure.

## Discussion

A positive family history of delayed emergence from anesthesia is a significant indicator for diagnosis, although it is often not present in cases where parental surgical history is unknown or both parents are heterozygotes for the *BCHE* mutation [2,3]. Aside from a comprehensive history, no routine screening tests are recommended unless the patient has a positive family history [2,3]. In this case, there was no known family history of delayed emergence. However, as a negative family history does not rule out this condition, it is recommended that these patients receive a plasma butyrylcholinesterase assay and inhibition testing. In cases of a positive family history, a pseudocholinesterase enzyme assay and/or dibucaine number is indicated [4]. Although a normal enzyme assay can be used to rule out deficiency, it cannot be used to distinguish between genetic and acquired causes of deficiency [3]. The dibucaine number, which measures the percentage of inhibition of enzyme activity after administration of local anesthetic (dibucaine), can be a useful adjunct to molecular genetic testing and initial screening to determine the root cause of the deficiency [9]. Normal dibucaine numbers are >70%, whereas 40-60% indicate partial inhibition, and <30% indicate minimal inhibition [9]. Values within the partial inhibition range indicate a heterozygous individual, and minimal inhibition is more indicative of homozygous abnormal individuals. In addition to dibucaine testing, the fluoride number can also be tested similarly, but it is less commonly used. Importantly, although enzyme activity is affected by acquired causes of pseudocholinesterase deficiency, these causes do not affect dibucaine or fluoride numbers [10]. These tests can be useful initial screening tools, but molecular genetic testing for *BCHE* mutations is considered the gold standard for making a definitive diagnosis [11].

When risk factors are identified through history and laboratory evaluation, anesthesia management should be individualized accordingly. Even partial inhibition on dibucaine testing can result in prolongation of neuromuscular blockade. Therefore, consideration of succinylcholine dose reduction or use of non-pseudocholinesterase-dependent neuromuscular blockers is indicated. These neuromuscular blocking agents fall under the non-depolarizing neuromuscular blocker category, such as cisatracurium, rocuronium, and vecuronium [12]. Additionally, the use of neuromuscular monitoring and the expectation of delayed emergence are also indicated. In patients who demonstrate minimal inhibition on dibucaine/fluoride number and/or have positive genetic testing, succinylcholine, mivacurium, and ester-based local anesthetics should be avoided entirely, and non-pseudocholinesterase-dependent neuromuscular blockers are indicated.

Of note, although not explicitly detailed in this case presentation, identifying a phase I versus a phase II block is also important in guiding the management of prolonged neuromuscular blockade. A phase I block is characterized by a gradual return of twitch height on train-of-four without fade, while a phase II block is associated with a fade on neuromuscular monitoring [13]. Unlike phase II blocks, phase I blocks cannot be treated with acetylcholinesterase agents such as neostigmine, as these agents can cause further respiratory compromise [13]. Pseudocholinesterase-deficient individuals may exhibit either phase, so phase identification is essential in guiding therapy [14].

This case details the appropriate postoperative management of a patient with delayed emergence from anesthesia due to a pseudocholinesterase deficiency. Due to the patient’s inability to metabolize the paralytic agents, the appropriate management includes supportive care (e.g., supplemental oxygen, maintaining normothermia) and continued mechanical ventilation until there is spontaneous recovery from neuromuscular blockade [15]. Although both deep and awake extubation are often routinely performed for pediatric adenotonsillectomy patients, no difference in respiratory adverse events has been recorded [16]. Therefore, either approach may be used in pseudocholinesterase-deficient individuals as long as neuromuscular recovery has been confirmed. There is no specific antidote for succinylcholine or mivacurium-induced paralysis in pseudocholinesterase deficiency, and acetylcholinesterase inhibitors (e.g., neostigmine) are ineffective in reversing depolarizing neuromuscular blockade. Therefore, quantitative neuromuscular monitoring, such as train-of-four stimulation, should be used to assess the degree of blockade. which can allow for appropriately timed extubation [17,18].

## Conclusions

Pseudocholinesterase deficiency, while rare, should remain on the differential in cases of delayed emergence after succinylcholine administration. Our case highlights the importance of perioperative vigilance, neuromuscular monitoring, and prompt recognition of unexpected, prolonged paralysis. It also details the importance of taking a thorough preoperative history to gauge risks of delayed emergence. Clear documentation and family counseling are essential to prevent recurrence and support safer perioperative anesthesia management.
